# Measuring and Predicting Individual Differences in Executive Functions at 14 Months: A Longitudinal Study

**DOI:** 10.1111/cdev.13217

**Published:** 2019-01-21

**Authors:** Rory T. Devine, Andrew Ribner, Claire Hughes

**Affiliations:** ^1^ University of Birmingham; ^2^ New York University; ^3^ University of Cambridge

## Abstract

This study of 195 (108 boys) children seen twice during infancy (Time 1: 4.12 months; Time 2: 14.42 months) aimed to investigate the associations between and infant predictors of executive function (EF) at 14 months. Infants showed high levels of compliance with the EF tasks at 14 months. There was little evidence of cohesion among EF tasks but simple response inhibition was related to performance on two other EF tasks. Infant attention (but not parent‐rated temperament) at 4 months predicted performance on two of the four EF tasks at 14 months. Results suggest that EF skills build on simpler component skills such as attention and response inhibition.

Children's ability to control their own thoughts and actions, or “executive function” (EF), has been a topic of intense interest in the field of developmental science for the past two decades (Carlson, Zelazo, & Faja, 2013). EF refers to a set of interrelated, domain general cognitive skills associated with the prefrontal cortex, namely: (a) the ability to override entrenched habits or impulses (or “inhibition”), (b) the capacity to update information held in mind (or “working memory”), and (c) the capability to switch between tasks (or “cognitive flexibility”; Friedman & Miyake, 2017). Research on the development, correlates, and consequences of EF has flourished in developmental science with the proliferation of child‐friendly measures (e.g., Carlson, 2005). Interest in EF is not surprising given that longitudinal studies demonstrate that normative individual differences in EF predict children's social understanding (e.g., Devine & Hughes, 2014), academic success (e.g., Blair, Ursache, Greenberg, & Veron‐Feagans, 2015), and behavioral adjustment (e.g., Schoemaker, Mulder, Dekovic, & Matthys, 2013). Although great progress has been made in understanding EF in early and middle childhood, relatively little attention has centered on individual differences in EF in children younger than 24 months. The overarching aim of the current study was to investigate the relations between and infant predictors of individual differences in EF in the second year of life.

## Measuring EF in the First 2 Years of Life

Pioneering work by Diamond and colleagues (Diamond, 1985, 1988) provided important early insights into the development of EF across the first 2 years of life. Diamond argued that infants’ completion of the A‐not‐B task (in which infants must locate an object hidden in one of two alternating locations) hinged on the ability to hold information about the object's location in mind (working memory) and overcome a prepotent response to reach toward a previously rewarded location (conflict inhibition; Diamond, 1985; Diamond & Doar, 1989). Both lesion studies in nonhuman animals (e.g., Diamond & Goldman‐Rakic, 1989) and neuroimaging studies in human infants (e.g., Bell, 2012) support the validity of these tasks as measures of frontal function. Across several small‐scale longitudinal studies, Diamond and colleagues demonstrated age‐related growth in performance across the A‐not‐B, the Delayed Response (a task structurally similar to the A‐not‐B but with a fixed trial order), and object retrieval tasks over the first 2 years of life (Diamond, 1985, 1988). These studies provided evidence for the early emergence of EF and demonstrated the feasibility of testing EF in the first 2 years of life. While ground breaking, these early findings did not focus on individual differences in EF. Although the results suggested similar patterns of growth across a range of EF tasks, these studies were based on single tasks and so the relations between these measures were not examined.

Mirroring trends in EF research on children aged 24 months and over (e.g., Ansell, Wouldes, & Harding, 2017; Carlson, 2005; Garon, Smith, & Bryson, 2014; Hughes & Ensor, 2005; Leve et al., 2013; Mulder, Hoofs, Verhagen, Van der Veen, & Leseman, 2014; Wiebe, Espy, & Charak, 2008), researchers have extended the task battery approach to research on infants aged between 12 and 15 months (Johansson, Marciszko, Brocki, & Bohlin, 2016; Miller & Marcovitch, 2015; Wiebe, Lukowski, & Bauer, 2010). These studies have combined EF tasks previously studied in isolation to construct task batteries that include: (a) response inhibition tasks in which infants refrained from touching an attractive toy (e.g., a glittery wand; Miller & Marcovitch, 2015; Johansson et al., 2016); (b) the A‐not‐B or similar tasks (Miller & Marcovitch, 2015; Wiebe et al., 2010); (c) the Three Boxes task in which infants searched for toys hidden in three distinct locations (Miller & Marcovitch, 2015; Wiebe et al., 2010); and (d) sorting tasks in which infants learned to sort objects according to one rule and then sorted the same objects according to a different rule (Johansson et al., 2016; Miller & Marcovitch, 2015). Single EF tasks are unlikely to provide a pure assay of a particular aspect of EF but are often organized in terms of a perceived primary EF demand (Garon, Bryson, & Smith, 2008). Tasks are selected to capture simple response inhibition (e.g., overcoming the tendency to touch an attractive toy in the attractive toy task), conflict inhibition (e.g., overriding the tendency to search in a previously rewarded location in the A‐not‐B), working memory (e.g., holding the location of an object in mind in the Three Boxes task), and flexibility (e.g., replacing a learned stimulus–response set with a new one in the sorting tasks; Garon et al., 2008).

High levels of task completion in each of these studies suggests that short task batteries can be used to study EF in children younger than 2 years of age. That said, small sample sizes (all three studies involved fewer than 66 infants) precluded investigation of both individual differences in task performance and the psychometric properties of EF tests designed for use in the first 2 years of life. Addressing these two gaps, we sought to test a relatively large sample and adopt a latent variable analytic approach, which offers two distinct advantages. First, item response theory (IRT) models allowed us to estimate the reliability of each EF test at different levels of ability (Asparouhov & Muthèn, 2016; Thissen, 2000). Second, confirmatory factor analysis (CFA) allowed us to assess the fairness of test items across different groups (e.g., boys and girls) to ensure that, once latent ability was taken into account, scores were not unduly influenced by group membership (Brown, 2015). CFA has been employed to examine the psychometric properties of EF tests in the preschool years (e.g., Willoughby, Blair, Wirth, & Greenberg, 2010; Willoughby, Wirth, & Blair, 2012), but has yet to be applied in infant studies of EF. Using a battery of tasks based on those reported in previous small‐scale studies, our first aim was to investigate the feasibility of measuring individual differences in EF in the second year of life and, for each task, examine both reliability and fairness.

## Relations Between Measures of EF in Infancy

As noted earlier, recent studies have adopted a task battery approach to studying EF in the first 2 years of life. These batteries have typically included tasks purported to place demands on simple response inhibition, conflict inhibition, working memory, and flexibility. Classical test theory postulates that if these diverse measures are indeed capturing individual differences in a common underlying ability, namely EF, then we would expect performance across tasks to be correlated despite the apparent differences between tasks (e.g., Carroll, 1978). Among children under 24 months old, Wiebe et al. (2010) reported no correlation between performance on the A‐not‐B task, and the Three Boxes task. Likewise, Miller and Marcovitch (2015) found no consistent evidence for cohesion among a battery of EF tasks including the A‐not‐B task, a delay task, the Three Boxes task, and a sorting task. Johansson et al. (2016) observed a similar pattern in that there was no consistent evidence for correlations across EF tasks including a box‐search task, a delay task, and sorting task.

These findings stand in clear contrast with larger studies examining the correlations between measures of EF in toddlerhood (e.g., Hughes & Ensor, 2005) and the preschool years (e.g., Willoughby et al., 2010). Such studies typically show moderate associations between tasks and evidence for a single latent factor underpinning individual differences in performance across diverse EF tasks (e.g., Wiebe et al., 2008). Comparing these cross‐sectional results with data from adults, researchers have argued that the functional organization of EF changes across development, emerging first as a unitary ability before fractionating into more specialized abilities in adulthood (e.g., Best & Miller, 2010; Friedman & Miyake, 2017; Miyake & Friedman, 2012; Wiebe et al., 2011).

There are at least two possible explanations for the discrepancy between infant and preschool studies of performance on EF task batteries. From a methodological standpoint, the small samples employed in the studies of infant EF may have limited the power to detect modest associations between measures. From a conceptual standpoint, the absence of correlations among EF tasks in the first years of life could reflect the fact that EF emerges first as separable component skills that only become more integrated and co‐ordinated as infants mature (Garon et al., 2008). Tasks placing demands primarily on the ability to hold information in mind (e.g., the Three Boxes task) would therefore not be expected to correlate strongly with tasks placing considerable demands on overriding a prepotent response or shifting to a new stimulus–response set (e.g., A‐not‐B, sorting tasks). However, Garon et al. (2008) argued that simple response inhibition, the ability to withhold or delay a response, is a foundation for later emerging complex EF skills. Even in the absence of relations between other measures of EF, simple response inhibition might therefore correlate with tasks that place demands primarily on other aspects of EF. Supporting this hypothesis, Johansson et al. (2016) and Miller and Marcovitch (2015) have reported weak‐to‐moderate correlations between infant performance on a prohibition task and sorting tasks. Moreover, performance on prohibition tasks in the first 3 years of life predicts EF at age 17 (Friedman, Miyake, Robinson, & Hewitt, 2011). Extending this existing work, our second aim was to examine the correlations between different EF tasks in a large sample of 14‐month‐old children.

## Predictors of EF During the Second Year of Life

Although few studies have investigated variation in EF in the first 2 years of life, other potentially related dimensions of individual differences, such as attention and child temperament, have been extensively studied in this developmental period (e.g., Hendry, Jones, & Charman, 2016). Research on infant visual attention, as measured by simple habituation paradigms, indicates that infants vary considerably in the extent to which they spend time looking at novel stimuli and that this variation appears to be moderately reliable over time (Bornstein & Colombo, 2015). Longer (or “sticky”) looking times toward new stimuli between the ages of 2 and 7 months are posited to indicate inefficient information processing (Bornstein & Colombo, 2015). That is, longer looking times are the result of less efficient strategies of encoding new information from the environment. Supporting this view, ample evidence now demonstrates infant looking times in the first months of life moderately predict general intelligence later in childhood (Bornstein, 2014; Kavsek, 2004).

Alongside information processing, infant looking times during attention tasks could also capture emerging executive control. That is, infants who spend longer looking at a novel stimulus in their environment may be less able to exert control over what aspects of the environment they attend to. Being able to disengage from an environmental stimulus is a hallmark of EF (Russell, 1996). Infant attention has recently been highlighted as an important precursor of EF (Garon et al., 2008; Hendry et al., 2016). Supporting this account, a study has shown that longer looking times (measured at 5 months) are negatively correlated with EF in early childhood (Cuevas & Bell, 2014). Researchers have yet to investigate whether variation in visual attention in the first 6 months of life predicts more proximal indicators of EF in the second year of life. We therefore sought to investigate the relations between individual differences in infant looking times at 4 months and performance on EF tasks 10 months later.

Temperament, defined as variation in reactivity to the environment and regulation of emotion and behavior (Rothbart, Ahadi, Hershey, & Fisher, 2001), is another widely studied dimension of difference in infancy. Temperamental traits have traditionally been studied in isolation from EF but the past decade has witnessed efforts to unite these once disparate bodies of scholarship (e.g., Blair & Raver, 2015; Nigg, 2017). Aspects of temperament, in particular effortful control (i.e., the regulation of motor behavior, attention, and emotion) and negative affect (i.e., poor emotional regulation in response to changes in the environment), are conceptually overlapping with EF (Hendry et al., 2016; Liew, 2012; Nigg, 2017). Indeed, evidence from early childhood indicates that there are moderate links between effortful control and children's performance on measures of EF but that ratings of effortful control and EF show unique associations with academic outcomes (e.g., Blair et al., 2015).

Hendry et al. (2016) have suggested that infant temperamental traits might predict later EF. Specifically, infants who exhibit greater levels of control over their behavior (e.g., interacting with objects for prolonged periods) and lower levels of negative affect in response to changes in the environment might be better able to control their thoughts and actions than infants who lack the ability to control their responses to and engagement with the environment. Infant negative affect and duration of orienting (i.e., attending to and interacting with objects for long durations) show relative stability over time and predict negative affect and effortful control, respectively, in early childhood (e.g., Putnam et al., 2008). Researchers have yet to investigate the links between infant temperament during the first year of life and EF task performance during the second year of life. We therefore examined the associations between parental ratings of infant duration of orienting and negative affect at 4 months and later EF at 14 months. In summary, our third aim was to examine whether attention and temperament at 4 months predicted individual differences in later EF performance.

## Summary of Aims

Our longitudinal study had three distinct aims. Our first aim was to examine the feasibility of measuring individual differences in EF in the second year of life using a battery of tasks and to examine the reliability and fairness of each of these tasks. Our second aim was to examine the relations between different measures of infant EF at 14 months. Our third aim was to examine the longitudinal associations between attention and temperament (i.e., negative affect and duration of orienting) at 4 months and EF at 14 months.

## Method

### Participants

We now report how we determined our sample size, all data exclusions, all manipulations, and all measures in the study. The data were taken from a larger international study of maternal and paternal influences on children's self‐regulation and adjustment in the first 2 years of life and have not been reported elsewhere. The sample size was determined a priori as part of the larger project to investigate paternal and maternal influences on children's self‐regulation in the first 2 years of life. Allowing for 10% attrition, a sample of 210 participants would provide 80% power to detect medium effects (*f* ^2^ = .15) at the .01 level of significance in regression analyses with up to 10 predictors (Faul, Erdfelder, Lang, & Buchner, 2007).

We recruited 213 expectant mothers and fathers attending antenatal clinics and ultrasound scans at a regional maternity hospital in the East of England. To be eligible participants had to be: (a) first‐time parents, (b) living together at the time of their child's birth, (c) expecting delivery of a healthy singleton baby, (d) planning to speak English as a primary language with their child, and (e) have no history of severe mental illness (e.g., psychosis) or substance misuse. An additional eight families were recruited but were ineligible for follow‐up due to birth complications or having left the country. Of these families, 196 (92%) agreed to participate in a home visit when their infants (109 boys, 87 girls) were 4 months old, *M*_age_ = 4.12 months, *SD* = 0.39 months, range: 2.97–5.63 months. At follow‐up, two further families declined to take part and one family that missed the 4‐month visit participated at 14 months. This meant that 195 families took part when their infants (108 boys, 87 girls) were 14 months old, *M*_age_ = 14.42 months, *SD* = 0.59 months, range: 13.10–18.40 months. There were three outliers aged over 16 months but 98.5% of the infants were aged between 13.10 and 15.83 months. We retained these cases to maximize our sample size. The sample was predominantly White British: 92.8% of mothers and 94.9% of fathers. The majority of mothers (84.6%) and fathers (77%) had an undergraduate degree or higher. Mothers (60.8%) and fathers (61.4%) were drawn mainly from professional occupations.

### Procedure

The study protocol was approved by the National Health Service (UK) Research Ethics Committee. Expectant parents completed an online questionnaire and in‐person interview approximately 1 month before their due date. The families were then contacted to participate in a 4‐month follow‐up home visit. Each family completed two short home visits at 4 months lasting approximately 30 min in duration. We used home visits over laboratory visits to increase the uptake of our study and to ensure that infants were not distracted by unfamiliar environments. Each visit consisted of three short parent–child observations (not described here), parental questionnaires, and a parental interview. The infant completed the attention task at the first visit. The families were contacted approximately 10 months later (*M* = 10.30 months, *SD* = 0.65, range: 8.34–14.67 months) to participate in a follow‐up visit. Each family completed one home visit lasting approximately 1 hr. These visits consisted of three short parent–child interactions (not discussed here), parental interviews, and a short testing session (lasting approximately 10 min) with each child. EF tasks were administered in a fixed order: Prohibition task, Three Boxes task, Ball Run task, Delayed Response task.

### Measures

#### Attention at 4 Months

In the *Attention* task (Cuevas & Bell, 2014), infants were seated on a parent's lap facing the examiner (seated approximately 1 m from the infant). Using a “Whoozit Baby's Friend” toy, the examiner rattled the stimulus three times and held it up to his/her right or left (counterbalanced across infants). The examiner held the stimulus in position until the infant looked away for at least 3 s. At this point, the examiner lowered the toy and repeated the procedure for three further trials. Gaze was recorded using a camera on a tripod behind the examiner. The footage was coded offline using JHab Java Habituation Software (Version 1.0.0; Casstevens, 2007). We recorded the amount of time spent looking at the stimulus on each trial. Inter‐rater reliability based on 45 cases was acceptable, for all four trials .77 < intraclass correlation (ICC) < .98. We calculated children's median looking duration across the four trials of the task (Cuevas & Bell, 2014).

#### Temperament at 4 Months

Mothers and fathers completed the *Brief Infant Behavior Questionnaire* (Putnam, Helbig, Garstein, Rothbart, & Leerkes, 2014) prior to the 4‐month home visit to provide a measure of infant temperament. Parents were asked to rate the frequency of behaviors ranging from 1 (*never*) to 7 (*always*) across seven items relating to infant Distress to Limitations (e.g., crying or fussing when left in a crib) and six items relating to infant Duration of Orienting (e.g., playing with a toy for 5–10 min). Items from each scale were averaged across mothers and fathers to create two uncorrelated scores representing Distress to Limitations (α = .76) and Duration of Orienting (α = .75). In both cases, higher scores indicated a greater frequency of the reported behaviors.

#### EF at 14 Months

Infants completed a short battery of four tasks (lasting approximately 10–12 min) based on those previously reported (Johansson et al., 2016; Miller & Marcovitch, 2015). Infants were seated on a parent's lap across a table from the examiner. Parents were advised to remain silent during each task and not to influence their infant's behavior through gesture or vocalization. Infants were monitored carefully and provided with breaks between tasks if necessary. Infants were praised at the end of each task regardless of performance to maintain their interest in participating. We obtained multiple indicators of performance from each task to allow us to adopt a latent variable approach to analyzing task performance (e.g., Willoughby et al., 2010).

In the *Prohibition task* (Friedman et al., 2011), infants were required to resist touching an attractive toy. The examiner showed the infant a shiny glitter wand (“Mystic Glitter Wand”) and attracted the infant's attention verbally for up to 15 s. Before placing the wand down within the infant's reach, the examiner looked the infant in the eye and raised their index finger in a prohibitive manner and said: “No, don't touch!”. The examiner then turned around for 30 s from when the wand was released. We recorded the latency to first touching the wand following the placement of the wand in front of the infant (possible range: 0–30 s). Double‐coding of 60 of videos revealed high inter‐rater agreement, ICC = .99, *p *<* *.001. Scores were bi‐modally distributed and were collapsed into two categories (i.e., 0 = touches before 30 s; 1 = does not touch before 30 s).

In the *Three Boxes* task (Miller & Marcovitch, 2015), infants were required to find three toy cars (i.e., red, yellow, and blue plastic cars) hidden in three toy garages with colored doors (i.e., red, yellow, and blue; “Ambi Toys Lockup Garage”) with a short delay of 5 s between each search. The examiner introduced the infant to three toy cars and allowed the infant to explore these briefly. The garage toy was placed opposite the infant just out of reach. The examiner attracted the infant's attention as each car was placed into a garage (i.e., the blue car in the blue garage, the yellow car in the yellow garage, and the red car in the red garage). The examiner then closed the three doors simultaneously and then picked up a white card (29.7 × 42 cm) to block the infant's view of the garages, counting out loud for 5 s while looking at the child. The white board was removed and the infant was encouraged to retrieve a car by pointing to a door. Since all garages contained a car, the infants were always successful on the first trial. The examiner praised the infant and allowed the infant to play briefly with the car. The retrieved car was taken from the infant and the examiner showed the infant that the car was being placed in a bag behind the examiner. The examiner pointed to the empty garage and closed the door before proceeding to the next trial. For each subsequent trial, infants were praised and allowed to handle the retrieved car when they were successful. If infants pointed to an empty garage, the examiner opened the garage, looked inside and said “Oh, it's not there. Let's have another go” and reclosed the door before starting the next trial. The task continued until the infant retrieved all three cars or until the infant made three consecutive errors. Scoring took place offline and double‐coding of 60 videos revealed perfect inter‐rater reliability for each trial, κ = 1.00. In scoring this task, we sought to capture data about the efficiency of children's searching strategies. Simple records of the numbers of cars retrieved would not provide any data on the efficiency with which children retrieved the cars. We reasoned that children with high levels of working memory and inhibitory control would find cars more efficiently than their peers. We therefore adopted a scoring procedure devised by Garon et al. (2014) in a similar multilocation search task and created two scores: total number of searches to find the (a) second and (b) third cars (i.e., 0 = did not find; 1 = 3 searches; 2 = 2 searches; 3 = 1 search). We devised a third score, based on adult research on self‐ordered search tasks (Owen, Downes, Sahakian, Polkey, & Robbins, 1990), to measure children's use of an efficient search strategy. The strategy score recorded the approach used to search for the hidden cars with higher scores indicating a more efficient search strategy (i.e., 0 = starts in the middle, 1 = starts at either edge, 2 = starts at either edge and then selects middle but then repeats a search, 3 = starts at edge, then middle, then other edge).

In the *Delayed Response* task (Diamond & Doar, 1989), infants were required to find an attractive toy hidden in one of two locations after a fixed five‐delay. The Delayed Response task is similar in structure to the A‐not‐B task and shows a similar developmental trajectory (Diamond & Doar, 1989). We opted for this task over the A‐not‐B as the fixed trial order made it possible to scale‐up the task for a large‐scale study of individual differences. The infant was introduced to a touch‐activated light‐up ball and allowed to handle the toy briefly. The examiner then placed the ball in one of two black plastic boxes spaced 30 cm apart (left or right, counterbalanced) and turned the two boxes over simultaneously to hide the ball. The examiner occluded the infant's view of the two boxes with a laminated white card (29.7 × 42 cm) and counted out loud to 5. When the card was removed, the infant was asked to find the ball. If successful, the infant was praised and permitted to handle the ball briefly. If unsuccessful, the examiner showed the empty box, saying “Oh no! It's not there!” and then revealed the true location of the ball and did not permit the infant to handle the ball. The infant completed eight trials in a fixed order depending on whether they started on the Left (L) or Right (R): L, L, R, R, L, R, R, L or R, R, L, L, R, L, L, R. Scoring took place offline and double‐coding of 60 videos revealed perfect inter‐rater reliability for each trial, κ = 1.00. We based the scoring procedure on Diamond and Doar (1989) and Espy, Kaufman, McDiarmid, and Glisky (1999) and derived three scores from this task: (a) number of trials passed before the first error (i.e., 0 = 0 trials; 1 = 1 trial; 2 = 2 trials; 3 = 3 trials; 4 = 4 or more trials); (b) number of errors in “repeat following error” trials (or perseverative errors), that is, when the object is hidden in same location after an unsuccessful retrieval (reverse coded; i.e., 0 = 2 perseverative errors; 1 = 1 perseverative error; 2 = no perseverative errors); and (c) number of correct reversals, that is, a correct reach after passing the preceding trial where the ball was hidden in the opposite location (i.e., 0 = none; 1 = 1 correct; 2 = 2 correct; 3 = 3 or more correct).

The *Ball Run* task was based on the Trucks Task developed by Hughes and Ensor (2005). Prior work has demonstrated that 12‐month‐old infants struggle with simple sorting paradigms even before a shift to a new rule is introduced (Johansson et al., 2016). Forming and maintaining a task set is itself considered to be an early precursor of EF (Garon et al., 2014). We sought to create a simple rule learning task built around an age‐appropriate toy. Infants were introduced to a specially adapted ball run toy (“Legler Hammer Ball Marble Run Preschool Learning Toy”). This toy had three circular holes on the top running from left to right (i.e., green, yellow, red) and a metal chute to allow a ball to roll down through the toy. The toy was fitted with a transparent plastic panel to cover the front of the toy (facing the infant) while allowing the back of the toy to be accessible to the examiner. Two metal brackets were fixed beneath the holes allowing the examiner to close two holes and open only one hole (i.e., green or red). The middle (yellow) hole remained sealed for the entire task. On the floor of the toy, we placed a switch activated speaker programmed to play 5 s of a nursery song (“The Wheels on the Bus”) when pressed. In the rule learning phase, the examiner introduced the toy to the infant by showing them how to activate the musical switch by placing either the green ball in the green hole (on the left‐hand side of the toy) or the red ball in the red hole (on the right‐hand side of the toy; counterbalanced across children). After this demonstration, the examiner handed the ball to the infant, directly over the middle of the toy and looking at the infant and said “Now you try!” Infants were praised for each correct placement and reinforced through activation of the musical switch. If the infant placed the ball onto the closed hole, the examiner took the ball from the infant and said “Oh, it didn't work!” and proceeded to the next trial. There were six test trials in this phase.

If the infant scored four or more trials correctly (*N* = 61), the examiner proceeded to the reversal phase. Before this phase, the examiner took the ball they were using in the learning phase (e.g., the green ball) and placed it in a bag in view of the infant. The examiner then retrieved a different ball (e.g., the red ball) and attracted the infant's attention while they proceeded to close the open hole (e.g., the green hole) and open the closed hole (e.g., the red hole). The examiner demonstrated the placement of the new ball into the newly open hole (on the opposite end of the toy) and cheered when the music played. The examiner handed the ball to the infant as before and completed a further six trials. Scoring took place offline and double‐coding of 60 videos revealed perfect inter‐rater reliability for each trial, κ = 1.00. Children received one point for each correct placement. Infants who did not place four or more balls correctly in the learning phase received a score of 0 on each trial of the reversal phase.

#### Control Variables

Parents completed the brief version of the *MacArthur Communication Development Inventory* (MCDI; Fenson et al., 1994) during the 14‐month visit to measure children's language skills. From this checklist of 90 words, we calculated a total score by summing together the number of words that each infant understood and said respectively, α = .96. Children understood more words (*M* = 27.87, *SD* = 15.83) than they could say (*M* = 4.86, *SD* = 4.81), *t*(172) = 21.24, *p *<* *.001, and only 9.4% of children (*N* = 16) had begun combining words. Parents completed the *Ladder of Subjective Social Status* (Singh‐Manoux, Adler, & Marmot, 2003) in which they indicated their placement on a 10‐rung ladder where the top denoted those with the best education, income, and employment and the bottom those with worst. Parents’ job titles were used to rank occupations into one of nine standard classifications (ONS, 2010). Parents also reported on their highest level of educational attainment. These three scores were weakly‐to‐moderately correlated with each other in both mothers, .24 < *r *<* *.39, and fathers, .28 < *r *<* *.45, and were aggregated by calculating the mean standardized score across the six indicators of parental socioeconomic status (SES), α = .66.

## Results

### Analytic Strategy

We conducted our primary analyses using a latent variable framework in *Mplus* Version 8 (Muthèn & Muthèn, 2017). We first analyzed performance across each of the tasks to ascertain the feasibility of using a short battery of tasks to measure EF in 14‐month‐old children. Next, we examined the reliability of each task latent factor at different levels of underlying latent ability before examining the associations between each measure at 14 months. We then used structural equation modeling to examine the longitudinal association between attention and temperament at 4 months and children's later EF at 14 months (while controlling for language ability, age, gender, and SES). We evaluated model fit using four criteria: a nonsignificant chi‐square; a root‐mean‐square error of approximation (RMSEA) of < .08; a comparative fit index (CFI) of > .90; and a Tucker–Lewis index (TLI) of > .90 (Brown, 2015). Given that our data contained a combination of categorical and non‐normally distributed continuous variables, we used a mean‐ and variance‐weighted least squares estimator (WLSMV; Brown, 2015; Kline, 2011). Table 2 shows the extent of item missingness for each task indicator. A missing value analysis revealed that the data were missing completely at random (MCAR), Little's, χ^2^(33) = 31.89, *p* = .52. All available data were used when estimating WLSMV models (Asparouhov & Muthèn, 2010).

### Performance on Measures of EF at 14 Months

Boys and girls in the study did not differ significantly on any of the measured characteristics reported in Table 1. Table 2 shows children's performance on each of the EF task indicators at 14 months. We obtained Prohibition Task data from 173 children (88.7%). Data loss arose from parental interference (e.g., preventing the infant from touching the toy) with task performance while the examiner's back was turned (*N* = 15), video corruption (*N* = 4), or task malfunction (*N* = 3; e.g., the table wobbling while the examiner's back was turned). We obtained complete data from 180 (92.3%) children on the Three Boxes task. Three children refused to participate in the task and the video was corrupted for one child. The remaining children participated but refused to perform more than three searches. We obtained complete data for the Delayed Response task from 172 children (88.2%). The majority of missing cases were explained by child refusal or fatigue (*N* = 11), video corruption (*N* = 1), or refusal to continue the task (*N* = 11). We obtained complete data for the Ball Run learning phase (performance on all six trials) from 161 cases (82.6%). Note that 171 cases completed Trials 1 through 4. The task was not administered to three children due to fatigue or fussiness and data from one participant were missing due to video corruption. Missing data were due to refusal to continue the task beyond the demonstration trial (*N* = 10).

**Table 1:** Sample Characteristics

	*M*	*SD*	Range	*M* (*SD*)	
				Boys	Girls
Mother age (years)	32.61	3.59	25.10–43.15	32.61 (3.62)	32.59 (3.59)
Father age (years)	33.98	4.34	24.05–49.63	33.99 (4.46)	33.97 (4.23)
Mother ladder	7.32	1.39	3–10	7.17 (1.31)	7.51 (1.47)
Father ladder	7.32	1.27	4–10	7.31 (1.23)	7.33 (1.34)
Child age at T1 (months)	4.12	0.39	2.97–5.63	4.14 (0.40)	4.11 (0.39)
Child age at T2 (months)	14.42	0.59	13.10–18.40	14.46 (0.48)	14.37 (0.70)
T1 IBQ distress to limitations	3.67	0.88	1.71–6.10	3.65 (0.87)	3.71 (0.89)
T1 IBQ duration of orientation	4.26	0.87	2.09–6.92	4.27 (0.89)	4.25 (0.85)
T1 attention median look duration (s)	6.38	4.12	0.61–23.83	6.21 (3.86)	6.59 (4.44)
T2 MCDI total	32.73	18.56	3–80	32.45 (19.44)	33.06 (17.55)

Note

None of the gender contrasts were significant (all *t*s < 1.62). IBQ = Infant Behavior Questionnaire; MCDI = MacArthur Communication Development Inventory.

**Table 2:** Descriptive Statistics for Each Executive Function Task Indicator

Indicator	*N*	*M* (*SD*)	%Ceiling	*M* (*SD*)	
				Males	Females
Prohibition	173	0.36 (0.48)	35.8	0.37 (0.49)	0.35 (0.48)
DR perseverate	175	1.34 (0.59)	35.5	1.41 (0.58)	1.26 (0.61)
DR first error	175	1.81 (1.23)	12.6	1.81 (1.33)	1.79 (1.10)
DR reversals	172	0.69 (0.81)	2.9	0.80 (0.86)	0.55 (0.73)
Three boxes Car 2	190	1.51 (1.34)	35.5	1.36 (1.35)	1.69 (1.33)
Three boxes Car 3	180	0.47 (1.02)	11.7	0.52 (1.06)	0.41 (0.98)
Three boxes strategy	190	0.84 (0.73)	2.7	0.81 (0.69)	0.89 (0.78)
Ball Run Learn 1	181	0.44 (0.50)	44.2	0.50 (0.50)	0.38 (0.48)
Ball Run Learn 2	178	0.49 (0.50)	48.9	0.54 (0.50)	0.43 (0.49)
Ball Run Learn 3	172	0.44 (0.50)	43.6	0.45 (0.50)	0.42 (0.49)
Ball Run Learn 4	171	0.43 (0.50)	42.7	0.43 (0.50)	0.43 (0.50)
Ball Run Learn 5	161	0.45 (0.50)	45.3	0.48 (0.50)	0.41 (0.50)
Ball Run Learn 6	161	0.41 (0.50)	41	0.41 (0.50)	0.41 (0.50)
Ball Run Rev 1	169	0.12 (0.31)	12.4	0.12 (0.33)	0.13 (0.33)
Ball Run Rev 2	151	0.11 (0.31)	10.7	0.13 (0.33)	0.08 (0.28)
Ball Run Rev 3	169	0.14 (0.31)	14.2	0.15 (0.36)	0.14 (0.34)
Ball Run Rev 4	168	0.10 (0.29)	9.5	0.14 (0.35)	0.04 (0.20)
Ball Run Rev 5	163	0.15 (0.36)	15.3	0.14 (0.35)	0.17 (0.38)
Ball Run Rev 6	160	0.14 (0.35)	14.4	0.16 (0.37)	0.13 (0.34)

Note

None of the gender contrasts were significant (all *p*s > .01, *t*s < 2.11). DR = Delayed Response task; Ball Run learn = learning trials of the Ball Run task; Ball Run Rev = reversal trials of the Ball Run task.

Children were not at ceiling levels of performance on the Prohibition Task with just 35.8% of children waiting for the full 30 s. In the Three Boxes, the majority of children located the second car (59.5%) but far fewer children located the third car (19.4%). Most children started their search at the edge of the array (67.2%) but only 21.6% of these children followed this search by pointing to the middle (adjacent) box. In the Delayed Response task, most children (70.9%) got at least two trials correct before making their first error. Moreover, 40.7% of children made no perseverative errors on the Delayed Response task. Reversal trials proved challenging for the participants with 50.6% scoring 0 (when the performance on the previous trial was considered). Compared with performance on the four non‐reversal trials, *M* = 2.55, *SD* = 0.88, children obtained significantly lower summed scores on the reversal items, *M* = 0.70, *SD* = 0.84, *t*(158) = 23.57, *d* = 1.87. There was a strong effect of condition on the Ball Run task with performance on learning trials, *M* = 2.11, *SD* = 1.74, exceeding that on reversal trials, *M* = 0.75, *SD* = 1.48, *t*(168) = 10.73, *p *<* *.0001, *d* = 0.83.

### Measuring Individual Differences in EF Tasks at 14 Months

**Figure 1:**
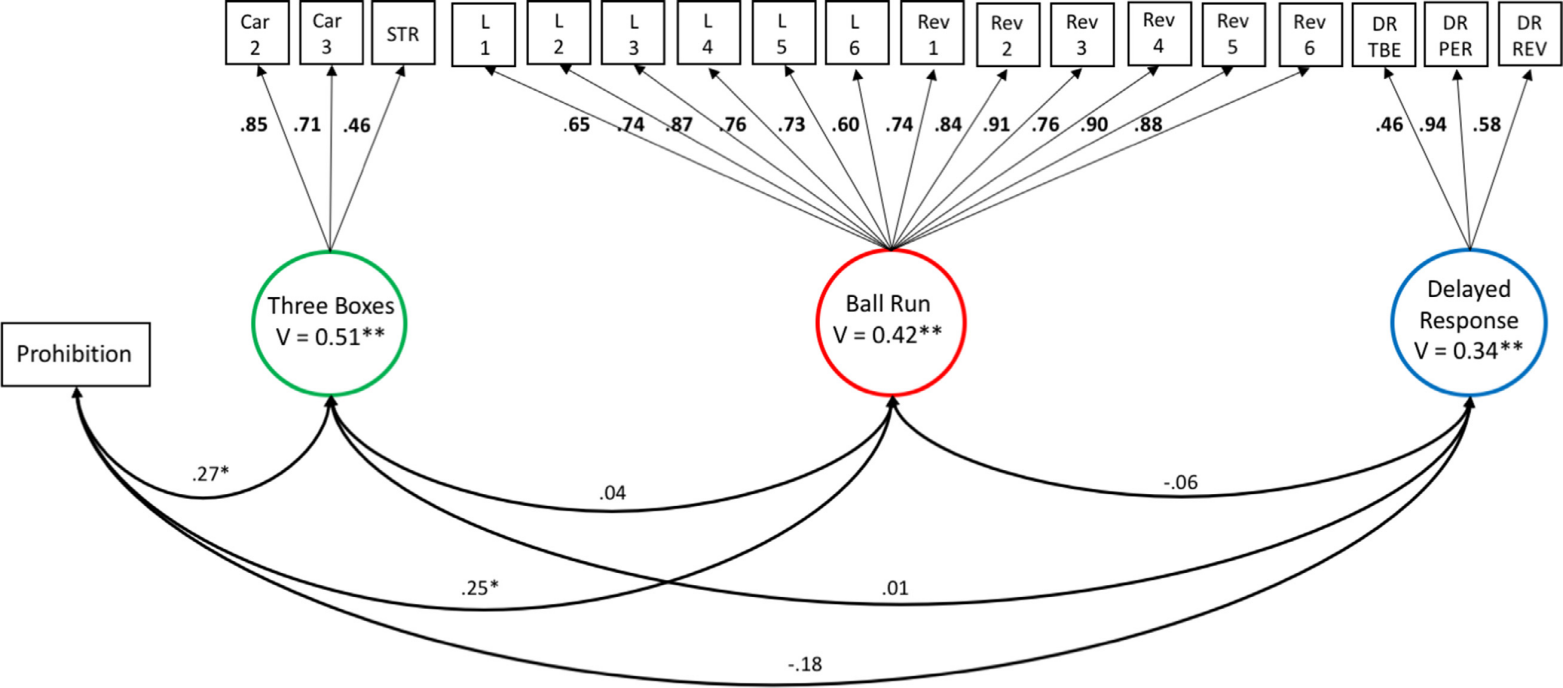
WLSMV standardized estimates for measurement model of executive function tasks at 14 months. *Note*. The error variances for each task indicator are omitted for ease of presentation. All factor loadings were significant at *p* < .01. WLSMV = mean‐ and variance‐weighted least squares estimator; V = latent factor variance; Car 2 = total number of searches to find Car 2 (reversed); Car 3 = total number of searches to find Car 3 (reversed); STR = strategy score; L = learning trials of the Ball Run task; Rev = reversal trials of the Ball Run task; DR = Delayed Response task; TBE = correct trials before first error; PER = perseverative errors (reversed); REV = total correct on reversal trials. *p < .05. **p < .01. [Color figure can be viewed at http://wileyonlinelibrary.com]

Table 3 shows the tetrachoric and polychoric correlations between each task indicator. Each of the tests included in the task battery were assumed to be unidimensional (i.e., these tests measured a single ability). We therefore tested a model in which each of the task indicators loaded onto separate latent factors representing the Three Boxes, Ball Run, and Delayed Response tasks. Note that since the Prohibition task was comprised of just one indicator, we did not estimate a latent factor for this task. Supporting the unidimensional structure of each EF task, this initial model provided a good fit to the data, χ^2^(147) = 165.08, *p* = .15, RMSEA = .025, 90% CI [.000, .043], CFI = .986, TLI = .984. Inspection of the modification indices revealed no evidence of local item dependence. Inclusion of a correlated error term (to reflect shared measurement effects) between the Car 2 and Car 3 indicators resulted in a nonpositive definite matrix and so the initial model was retained. The parameter estimates are shown in Figure 1. The variance for each task latent factor differed significantly from 0 indicating that there were marked individual differences in task performance on the Three Boxes, Est. = .51, *SE* = .16, *p* = .001; the Delayed Response, Est. = .34, *SE* = .10, *p* = .001; and the Ball Run, Est. = .42, *SE* = .11, *p *<* *.001.

**Table 3:** Tetrachoric and Polychoric Correlations Between Executive Function Task Indicators

		1	2	3	4	5	6	7	8	9	10	11	12	13	14	15	16	17	18
1	Prohibition	—																	
2	DR perseverate	.14	—																
3	DR first error	.12	.44**	—															
4	DR reversals	.12	.55**	.25**	—														
5	TB Car 2	.22**	.01	−.09	−.02	—													
6	TB Car 3	.24**	.11	.29**	.15*	.60**	—												
7	TB strategy	.07	−.08	−.18*	−.12	.28**	.25**	—											
8	Ball Run L 1	.21**	.02	−.05	−.10	.11	.09	.13	—										
9	Ball Run L 2	.11	.09	−.08	−.08	.21**	−.02	.06	.61**	—									
10	Ball Run L 3	.21**	.01	−.01	.03	−.03	−.11	−.05	.46**	.69**	—								
11	Ball Run L 4	.30**	−.01	.03	−.17*	.16*	−.11	.04	.49**	.60**	.71**	—							
12	Ball Run L 5	.23**	.11	.09	.08	−.01	.01	−.01	.60**	.64**	.75**	.57**	—						
13	Ball Run L 6	.20**	.19**	.12	.06	.02	−.03	.01	.41**	.39**	.62**	.66**	.53**	—					
14	Ball Run Rev 1	.05	.07	−.15*	.06	.16*	−.06	.01	.54**	.42**	.71**	.51**	.40**	.37**	—				
15	Ball Run Rev 2	.07	−.21**	−.10	−.07	.03	−.35**	.07	.47**	.61**	.58**	.43**	.48**	.23**	.67**	—			
16	Ball Run Rev 3	.21**	−.31**	−.18*	−.11	.16*	−.23**	.20**	.53**	.55**	.75**	.57**	.41**	.38**	.68**	.85**	—		
17	Ball Run Rev 4	.13	.21**	−.02	−.04	.03	−.05	−.02	.42**	.47**	.54**	.46**	.34**	.09	.72**	.70**	.74**	—	
18	Ball Run Rev 5	.25**	.13	−.02	−.06	.03	−.30**	−.11	.43**	.53**	.71**	.66**	.52**	.63**	.60**	.78**	.86**	.65**	—
19	Ball Run Rev 6	.25**	−.22**	−.09	−.27**	−.14	−.24**	.01	.47**	.57**	.63**	.58**	.56**	.28**	.58**	.78**	.82**	.80**	.86**

Note

Shaded gray cells indicate within task correlations. DR = Delayed Response task; TB = Three Boxes task;Ball Run L = learning trials of the Ball Run task; Ball Run Rev = reversal trials of the Ball Run task.

*p <* *.05. **p <* *.01.

**Figure 2:**
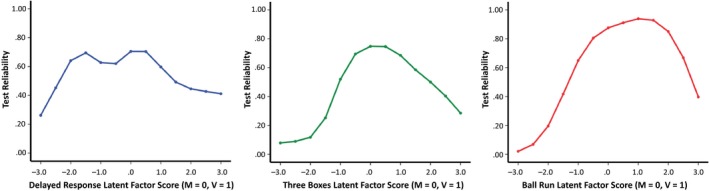
Measurement precision (reliability) as a function of ability for the Executive Function task latent factors at 14 months. *Note*. Approximate reliability coefficients (ρ) were computed from the total information curve for each task latent factor using the following formula: $\rho =1-\frac{1}{\mathrm{Inf}}$. [Color figure can be viewed at http://wileyonlinelibrary.com]

We next examined the precision of each task latent factor by generating a total information curve from a graded response IRT model. To this end, we freed the first factor loading of each latent factor and set the latent factor variances to 1 (to identify the model; Muthèn & Muthèn, 2017). Although maximum likelihood estimators have traditionally been used in IRT modeling, graded response models can be derived from categorical CFA using WLSMV (Asparouhov & Muthèn, 2016; Forero & Maydeu‐Olivares, 2009; Muthèn & Muthèn, 2017). As with standard IRT models based on maximum likelihood estimation, a total information curve for each latent factor can be estimated using WLSMV (Asparouhov & Muthèn, 2016). Information curves can be used to estimate the reliability of a test at a given level of ability (or theta; Thissen, 2000). If test information is high at a given level of theta, this means that a child with that level of ability can be measured with greater precision (Baker, 2001). The approximate reliability (ρ) of a test, ranging from 0 to 1, at a given level of theta can be estimated from the total information curve using the following formula: $\rho =1-\frac{1}{\text{Inf}}$, where *Inf* = test information (Embretson & Reise, 2000). Figure 2 shows the total information curves for each task latent factor. The *y*‐axis depicts the reliability co‐efficient based on the test information estimate. The Delayed Response provided reliable estimates (ρ > .70) in the average range of ability (0 < θ < 0.5). The Three Boxes task was most reliable (ρ > .70) in the average to above‐average range of ability (0 < θ < 1). The Ball Run was most reliable (ρ > .80) in the high range of ability (1 < θ < 2).

We used MIMIC (multiple indicators, multiple causes) modeling to examine the fairness of the EF tasks for use with boys and girls. Although measurement invariance is typically tested using multiple‐groups CFA (which can be used to test the invariance item loadings, item thresholds, error variances, and factor variances), we selected MIMIC modeling (which can be used to test for item threshold invariance) because of the uneven and relatively small group sizes within our sample (Brown, 2015). We tested a MIMIC model in which each latent task factor was regressed onto a dummy variable representing gender and each direct path between the test indicators and the gender variable was set to 0. Modification indices were inspected to determine if removing the constraint between an indicator and gender would result in improved model fit pointing to the presence of measurement non‐invariance (Brown, 2015). Measurement non‐invariance occurs when item performance is related to some characteristic other than the underlying latent ability (Brown, 2015). The model provided a good fit to the data, χ^2^(163) = 187.27, *p* = .09, RMSEA = .028, 90% CI [.000, .044], CFI = .982, TLI = .979, and inspection of the modification indices revealed no localized areas of strain. The model supported the assumption that gender had no direct effect on task performance (above the latent factor).

### Relations Between EF Tasks at 14 Months

Inspection of the standardized latent factor covariances (see Figure 1) revealed that there were weak associations between performance on the Prohibition task and the Three Boxes task and between the Prohibition task and the Ball Run task. Children who resisted touching the attractive toy performed better on both the Three Boxes task and the Ball Run task than those who touched the toy. There were no other significant correlations. The pattern of associations was similar when alternative scoring approaches were adopted (see Table S2).

### Early Predictors of EF Task Performance at 14 Months

We used structural equation modeling to examine the predictors of each EF task at 14 months. Table S1 contains a full correlation matrix for all manifest variables. To this end we specified a model in which each of the four 14‐month EF task latent factors and the Prohibition task indicator were regressed onto looking times from the attention task and ratings of Distress to Limitations and Duration of Orienting at 4 months. In addition, we controlled for variation in age, gender, language ability, and parental SES by regressing the 14‐month variables onto each covariate. This model provided (Table 4) a good fit to the data, χ^2^(258) = 281.29, *p* = .16, RMSEA = .02, 90% CI [.000, .037], CFI = .982, TLI = .977. The model accounted for 24.3% of the variance in the Delayed Response latent factor, 7.6% of the variance in the Three Boxes latent factor, 5.5% of the variance in the Ball Run latent factor, and 3.4% of the variance in the Prohibition task.

**Table 4:** WLSMV Estimates for Predictors of Executive Function (EF) Task Performance at 14 Months

	14‐Month EF measures											
	Prohibition task			Delayed response			Three boxes			Ball run		
	Est.	*SE*	Std.	Est.	*SE*	Std.	Est.	*SE*	Std.	Est.	*SE*	Std.
Covariate												
Age	−.18	.15	−.10	.01	.10	.01	.08	.10	.06	.14	.09	.13
Gender	.06	.20	.03	−.22	.12	−.17	.14	.14	.09	−.11	.11	−.09
SES	.02	.02	.13	.03	.01	.25*	−.004	.01	−.03	−.002	.01	−.02
Language	.002	.01	.04	.005	.003	.14	−.005	.004	−.14	.004	.003	.13
4‐Month predictor												
Attention	−.02	.02	−.08	−.05	.02	−.34**	−.03	.02	−.18*	.01	.01	.08
Dur. Orient.	.04	.12	.03	−.05	.07	−.07	.09	.08	.11	−.07	.07	−.10
Dis. Limit.	.05	.11	.05	.06	.07	.09	.06	.08	.08	−.02	.06	−.03

Note

The complete model output is presented in Table S3. Est = unstandardized estimate; *SE* = standard error of unstandardized estimate; Std. = standardized estimate; Dur. Orient. = duration of orienting; Dis. Limit. = distress to limitations; SES = socioeconomic status.

*p <* *.05. **p <* *.01.

Several paths in this model are worthy of note (for full model output see Table S3). First, individual differences in looking times during the attention task at 4 months uniquely predicted performance on both the Delayed Response latent factor and the Three Boxes latent factor. That is, children who showed longer looking times at 4 months performed poorer on these two tasks at 14 months than children who showed shorter looking times. Second, the two dimensions of parent‐rated temperament at 4 months (i.e., Duration of Orienting and Distress to Limitations) were not uniquely associated with any of the EF task latent factors or the Prohibition task at 14 months. Likewise, infant median looking times on the attention task were not associated with concurrent Duration of Orienting, Std. Est. = .12, 95% CI [−.03, .27], *p* = .12, or Distress to Limitations, Std. Est. = −.09, 95% CI [−.24, .06], *p* = .22. Third, language scores from the MCDI were not uniquely associated with performance on any of the EF tasks at 14 months. Fourth, the association between performance on the Three Boxes latent factor and the Prohibition task, Std. Est. = .31, 95% CI [.07, .55], *p* = .012, and between the Ball Run latent factor and the Prohibition Task, Std. Est. = .24, 95% CI [.02, .47], *p *=* *.03, remained statistically significant even when covariates were considered.

## Discussion

Our results support the feasibility of using task batteries to study individual differences in EF in the second year of life, show that common EF paradigms exhibit acceptable levels of reliability in average to above‐average ability children, and indicate that EF tasks can be applied fairly to boys and girls. More substantively, our results shed new light on the associations between measures of EF at 14 months and identify early infant precursors of EF task performance in the second year of life. We now discuss the implications of our results considering potential limitations and suggesting future directions for research.

### Measuring EF in the Second Year of Life

To date, most studies of EF in children younger than 24 months have primarily adopted either small samples or used only isolated tasks. This approach has meant that, with some exceptions (e.g., Johansson et al., 2016; Miller & Marcovitch, 2015; Wiebe et al., 2010), individual differences have been largely overlooked in favor of documenting age‐related differences in task performance. Although this approach has pointed to the dramatic development of EF in the first 2 years of life (e.g., Diamond, 1985), a focus on individual differences in EF has been lacking. To address these limitations, we recruited a relatively large sample of children and administered a battery of tasks comprised of widely used paradigms in the field. The suitability of these tasks for studying infant EF was supported by high levels of compliance (ranging from 82% to 92%) and the absence of obvious ceiling effects. There was striking variation in task performance even though our sample was drawn from a narrow age and socioeconomic range.

To our knowledge, our study marks the first attempt to use latent variable modeling to examine the properties of commonly used paradigms for studying EF in children aged under 24 months. Modern measurement approaches, such as IRT, have been applied to EF tests in preschool children (Willoughby et al., 2010, 2012). Our analytic approach allowed us to examine how test reliability (or precision) varied as a function of underlying ability (Embretson & Reise, 2000). Willoughby et al. (2010) argued that understanding the varying reliability of EF tests permits researchers to select more appropriate tests of EF for specific populations (e.g., children with developmental disabilities). The tasks in our battery were most reliable when measuring children in the average to above‐average ability range. Future studies of children at risk of developmental disabilities (e.g., siblings of children with autistic spectrum disorder) and research incorporating more socioeconomically diverse children will elucidate the reliability of these measures in children of varying abilities.

Our CFA models provide initial evidence that our battery of EF tasks showed no gender bias indicating that performance on the paradigms used in this battery were not unduly influenced by gender. It is worth noting that our sample consisted primarily of typically developing children from relatively well‐educated, ethnically homogenous families. Our results provide a platform for further work investigating the fairness of these tasks in developmentally at‐risk children and children of more varied SES. This will extend our understanding of whether observed differences in EF between typically developing children and children with problem behaviors in early childhood (for meta‐analysis see: Schoemaker et al., 2013) are detectable early in infancy.

### Relations Between Measures of EF in the Second Year of Life

Unlike in later childhood (e.g., Willoughby et al., 2010), but consistent with previous studies of 12‐ to 15‐month‐old children using smaller samples, we found no evidence for consistent correlations between distinct measures of EF at 14 months. Our results also mirror the weak and inconsistent correlations between EF tasks in large studies of children aged between 20 and 37 months (Ansell et al., 2017; Mulder et al., 2014). It is therefore unlikely that the lack of correlations among EF tasks in initial studies (e.g., Johansson et al., 2016; Miller & Marcovitch, 2015) was due to their small sample sizes. Our models demonstrated that each task exhibited significant variance so it was also unlikely that limited task variance accounted for the lack of task cohesion. Consistent with earlier studies, performance on the Prohibition task was moderately correlated with performance on other tasks. Children who resisted touching the attractive toy performed better on the Three Boxes and Ball Run tasks.

One lean interpretation of our results is that the weak associations between these widely used paradigms designed to index aspects of EF indicates that these measures are neither valid nor reliable indicators of infant EF. More specifically, although these tasks appear to measure a construct that “looks like” EF, these measures simply capture infants’ proficiency in other non‐EF related cognitive domains (e.g., motor skills, language) or infant temperamental characteristics. Indeed, if these measures were reliable indicators of EF, then we would expect continuity in rank‐order performance across tasks. At least two factors make this interpretation unlikely. First, numerous studies support the validity of the tasks used in our battery. For example, Friedman et al. (2011) have reported that infant performance on the Prohibition task predicts scores on a battery of measures of EF at age 17. Likewise, lesion studies of nonhuman primates indicate the frontal cortex underpins performance on the Delayed Response and Three Boxes tasks (Diamond & Doar, 1989; Petrides, 1995). Second, the weak and nonsignificant correlations in our study challenge the view that our tasks simply capture infants’ proficiency in language or specific temperamental characteristics.

A second interpretation of our results is that EF task performance in infancy might be unduly influenced by specific task demands. Unlike the first interpretation of our results, this view does not dismiss the tasks as unreliable or invalid measures of EF. Assuming the tasks do indeed measure aspects of emerging EF, how can we account for the observed pattern of associations? Despite being structurally very similar tasks (i.e., both involve a 5‐s delay, both involve searching for a hidden object), the Three Boxes and the Delayed Response were uncorrelated. In contrast, despite being structurally dissimilar, the Prohibition task was correlated with both the Three Boxes and the Ball Run tasks. Turning to the first of these findings, although the Delayed Response and Three Boxes are thought to involve aspects of working memory (Garon et al., 2008), one task involves having to track one's own searches for objects that do not change locations (i.e., the Three Boxes) and the other involves having to track an object hidden by another person that changes locations (i.e., the Delayed Response). Moreover, the initial trials of the Delayed Response are designed to set up a prepotent response and, conceivably, this must be overridden when searching for the toy. Although, both tasks might involve holding information in mind, the demands of each task are quite different. Turning to the tasks correlated with the Prohibition task, the associations with the Three Boxes and Ball Run could indicate that children who did not resist touching the wand were behaving impulsively when asked to retrieve a car in the Three Boxes (and failing to recall where they had previously searched) and when they were asked to place the ball in the Ball Run toy (failing to recall where the examiner had placed the ball at the start of the task). The pattern of observed associations could indicate that task features (and the demands placed on other nonexecutive aspects of perception, cognition, language, and motor skills) might exert a stronger influence on performance when EF is still emerging.

Along similar lines, Hendry et al. (2016) argued that since the first years of life are a period of substantial change, EF is not yet a stable characteristic. Therefore, other nonexecutive processes might interfere with task performance more so than in the preschool years (Hendry et al., 2016). That is, the so‐called “task impurity” problem may be more pronounced because specific task demands (e.g., for understanding verbal instructions) may mask infants’ emerging EF abilities (Clark et al., 2016). Although this “task demands” interpretation is plausible, as mentioned previously, our results indicated no consistent association between language ability and performance on each EF task.

A third interpretation of our results is that the absence of consistent correlations between different EF tasks reflects shifts in the functional organization of EF across early childhood. Specifically, EF may first emerge as separable component skills that become more coordinated, integrated, and controlled in the preschool years (Cuevas, Rajan, & Bryant, 2017; Garon et al., 2008). Indeed, comparisons of cross‐sectional factor‐analytic research on EF task performance from preschool to middle childhood, adolescence, and adulthood are leveraged to support the view that EF emerges first as a unitary ability before fractionating later in development (e.g., Wiebe et al., 2011). However, this interpretation faces at least two challenges. First, like other studies of young children (e.g., Wiebe et al., 2011; Willoughby et al., 2012), to minimize fatigue we did not include the multiple measures needed to test the separability of three EF components (e.g., Friedman et al., 2011). Second, developmental claims about the changing functional organization of EF require direct comparisons of the factor structure of EF in the same sample across an extended period in childhood. An important challenge for future research is therefore to determine whether or not the functional organization of EF changes from infancy through to the preschool years (and beyond).

A fourth developmental account of our findings rests on the claim that children's representational capacities underpin the emergence of EF in early childhood (e.g., Perner, 1998). Representational capacity permits children to hold information (e.g., stimulus–response pairings, prior actions, rules, goals) in mind and later integrate or distinguish between conflicting information. This capacity may only come online between the ages of 2 and 3 years and could explain the observed associations between EF tasks in the preschool years (Garon et al., 2008; Lang & Perner, 2002). Our results are consistent with this hypothesis. First, only 36% of the 14‐month‐old children in our sample performed at above‐chance levels on the learning phase of the Ball Run task (4/6 trials correct). This phase of the task involved forming a representation of a simple rule (i.e., the green ball goes in the green hole) while ignoring distractors (e.g., the red hole). Second, only 37% of children located the second car in the Three Boxes task with one search. Locating the car require children to hold in mind one previous search after over 5‐s delay. The late emergence of children's representational capacity may explain the weak and nonsignificant correlations between tasks that place demands on children's ability to hold information in mind (i.e., Ball Run, Delayed Response, and Three Boxes).

Note that the Ball Run and the Three Boxes tasks were correlated with the Prohibition task (but not each other). The association between Prohibition task performance and other measures of EF in our study, and in prior research (Johansson et al., 2016; Miller & Marcovitch, 2015), is in line with the view that infants rely on early‐emerging aspects of EF, such as simple response inhibition, when performing different EF tasks in the absence of more complex EF skills (such as updating or shifting; Garon et al., 2008). This hierarchical account of EF development (Cuevas et al., 2017; Garon et al., 2008) suggests that emerging forms of EF (e.g., response shifting) build on simpler component skills such as attention and response inhibition, rather than developing in parallel. For example, before being able to update information, a child must first be able to attend to the relevant and ignore the competing stimuli and then be able to hold or represent information in mind. Successful performance on measures such as the Three Boxes or Ball Run tasks might therefore require both inhibition *and* representation (e.g., Kloo, Perner, & Giritzer, 2010). Without the capacity to represent past actions, future goals or rules, children rely on basic inhibitory processes.

The lack of any clear association between performance on measures of EF and language ability appears to challenge the representational account. As noted by Perner and Dienes (2003), children's representational capacity underpins the emergence of productive, nonroutine word combinations (at around 24 months of age), but are much less likely to be implicated in children's first words, which typically emerge around 13 months of age as part of social routines. From this perspective, it is worth noting that the children in our study were, on average, just 14 months old and parents reported that only 9.4% of the sample had started to combine words. Given that children's language use becomes increasingly representational in nature with age, we would expect to see stronger associations between nonroutine productive language and EF around 24 months (Perner & Dienes, 2003). An obvious goal for future work would be to test this hypothesis within a longitudinal study.

### Predictors of EF in the Second Year of Life

To our knowledge, our study was the first to investigate the association between individual differences in visual attention measured in early infancy and performance on EF tasks measured in the second year of life. Our results complement prior research showing that infants with long looking times, measured using simple visual habituation tasks, perform poorly on EF measured in the preschool years (Cuevas & Bell, 2014). Specifically, infants with longer looking times performed worse than those with shorter looking times on two of four of the EF tasks at 14 months (even when individual differences in language ability, age, gender, and parental SES were considered). Infants’ looking times were related to performance on the Three Boxes and Delayed Response tasks but not the Prohibition or Ball Run tasks. Infant looking time measures taken in the first months of life are thought to index the efficiency of information processing (Bornstein & Colombo, 2015). This view is supported by a body of research showing moderate correlations between infant looking times and measures of IQ later in childhood (Kavsek, 2004). Rather than being an early indicator of executive control or precursor to EF (Hendry et al., 2016), infant looking times might be a domain general indicator of cognitive performance. That said, our results and those reported elsewhere (Cuevas & Bell, 2014) suggest that there are unique associations between infant looking times and later EF over and above general verbal ability. This raises the possibility that infant visual attention might predict later general intelligence via its association with EF (Cuevas & Bell, 2014). Future studies examining the unique associations between infant looking times and later IQ and EF are needed to test this hypothesis.

Our study also marks the first attempt to examine the relations between early‐emerging dimensions of infant temperament, namely negative affect (as measured by Distress to Limitations) and alertness/orientation (measured by Duration of Orienting), and performance on EF tasks in the second year of life. Despite evidence demonstrating associations between aspects of temperament (e.g., effortful control) and EF in older children (e.g., Blair et al., 2015), we found no concurrent links between temperament traits and infant looking times at 4 months or longitudinal associations between temperament traits and later EF at 14 months. It is worth noting that our study participants were all first‐born infants, such that the reliability of temperament ratings is open to question as parents may not have a meaningful yardstick against which to rate their child's behavior. That said, we used two informants for every child and their ratings were significantly correlated. Even if EF tasks at 14 months do not measure stable underlying traits (as discussed earlier), individual differences in infant looking time measures are reliable (Bornstein & Colombo, 2015).

Our findings challenge the view that EF tasks and behavioral ratings of effortful control are united by a core dimension of inhibitory control (Liew, 2012) and that high levels of negative affect (an indicator of reactivity) are a risk factor for EF problems (Hendry et al., 2016). Despite conceptual overlap (e.g., Nigg, 2017), ratings of effortful control and negative affect capture behaviors that may not require EF (e.g., “warming up” to an unfamiliar adult or being content to be left alone in a crib). Likewise, EF is not always employed for the purposes of self‐regulation of emotions or behavior. For instance, performance on EF tasks is related to mathematics performance, whereas ratings of effortful control are not (Blair & Razza, 2007). Investigations of the relations between EF and task‐based measures of effortful control and negative affect (e.g., Planalp, Van Hulle, Gagne, & Goldsmith, 2017) are needed.

### Limitations

Before concluding, it is worth reiterating two key limitations of our study. First, our sample consisted of first‐time parent families from relatively affluent backgrounds. This study feature may explain the weak and nonsignificant links between SES and EF and between temperament and EF. The weak association between SES and task performance could reflect the limited variance in SES within our sample. In addition, first‐time parents may not provide accurate ratings of their children's behavior on temperament questionnaires as they may not have clear reference points. Second, to minimize fatigue among participants, we could not administer multiple measures of each hypothesized component of EF. While this approach is typical in infant and early childhood research on EF (e.g., Willoughby et al., 2010), it meant that task‐specific variance and true‐score variance (i.e., representing a hypothesized component of EF) could not be distinguished. Future studies should incorporate multiple measures of each hypothesized EF component to separate genuine between‐person differences in EF from task‐specific variance.

### Conclusions

Notwithstanding the acknowledged limitations, our results contribute to the study of individual differences in EF in several ways. At a methodological level, our data from a large sample of infants support the feasibility of using a battery of EF tasks to measure variation in EF performance and indicate that the tasks are reliable measures for children in the average range of ability. From a theoretical perspective, children's poor representational capacities may account for the limited associations among measures of EF in the second year of life. Our results support the view that EF skills build on simpler component skills by showing that (a) early indicators of attention at 4 months (but not dimensions of temperament) predict performance on measures of EF at 14 months and (b) simple response inhibition may underpin performance on a range of different EF tasks at 14 months. These findings lay the foundations for future research on individual differences in EF downward into infancy.

## Supporting information


**Table S1.** Correlation Matrix for All Manifest Variables
**Table S2.** Correlations Between Alternative Indicators of Executive Function Task Performance at 14 Months
**Table S3.** WLSMV Estimates for Longitudinal Model Predicting Executive Function Task Performance at 14 Months
