# Supplementary material for: Measuring and Predicting Individual Differences in Executive Functions at 14 Months: A Longitudinal Study
Source: Child Dev. 2019 Jan 21;90(5):e618–36. doi: 10.1111/cdev.13217 (PMC6849706; doi:10.1111/cdev.13217)
Supplement: Supplementary file 1 — Table S1. Correlation Matrix for All Manifest Variables Table S2. Correlations Between Alternative Indicators of Executive Function Task Performance at 14 Months Table S3. WLSMV Estimates for Longitudinal Model Predicting Executive Function Task Performance at 14 Months [file CDEV-90-e618-s001.docx]

**Measuring and Predicting Individual Differences in Executive Functions at 14 Months: A Longitudinal Study.**

**Supporting Information for Online Publication**

Table S1. Correlation Matrix for All Manifest Variables.

Table S2. Correlations between Alternative Indicators of Executive Function Task Performance at 14 Months.

Table S3. WLSMV Estimates for Longitudinal Model Predicting Executive Function Task Performance at 14 Months.

Table S1. *Tetrachoric, Polychoric and Pearson Correlations for All Manifest Variables.*

|  | 1 | 2 | 3 | 4 | 5 | 6 | 7 | 8 | 9 | 10 | 11 | 12 | 13 | 14 | 15 | 16 | 17 | 18 | 19 | 20 | 21 | 22 | 23 | 24 | 25 | 26 |
| --- | --- | --- | --- | --- | --- | --- | --- | --- | --- | --- | --- | --- | --- | --- | --- | --- | --- | --- | --- | --- | --- | --- | --- | --- | --- | --- |
| 1 Age T2 | - |  |  |  |  |  |  |  |  |  |  |  |  |  |  |  |  |  |  |  |  |  |  |  |  |  |
| 2 Gen | -.07 | - |  |  |  |  |  |  |  |  |  |  |  |  |  |  |  |  |  |  |  |  |  |  |  |  |
| 3 SES | .11 | .09 | - |  |  |  |  |  |  |  |  |  |  |  |  |  |  |  |  |  |  |  |  |  |  |  |
| 4 Lang | .17 | .03 | -.07 | - |  |  |  |  |  |  |  |  |  |  |  |  |  |  |  |  |  |  |  |  |  |  |
| 5 D.O. | .07 | -.01 | -.04 | .16 | - |  |  |  |  |  |  |  |  |  |  |  |  |  |  |  |  |  |  |  |  |  |
| 6 D.L. | .06 | .03 | .15 | .14 | -.05 | - |  |  |  |  |  |  |  |  |  |  |  |  |  |  |  |  |  |  |  |  |
| 7 Attn | -.05 | .05 | .05 | .08 | .12 | -.09 | - |  |  |  |  |  |  |  |  |  |  |  |  |  |  |  |  |  |  |  |
| 8 Pro | -.07 | -.03 | .12 | .02 | .02 | .07 | -.06 | - |  |  |  |  |  |  |  |  |  |  |  |  |  |  |  |  |  |  |
| 9 DR P | .05 | -.13 | .16 | .09 | -.13 | .15 | -.19 | -.15 | - |  |  |  |  |  |  |  |  |  |  |  |  |  |  |  |  |  |
| 10 DR F | .04 | -.01 | .16 | .18 | -.02 | .11 | -.21 | -.12 | .44 | - |  |  |  |  |  |  |  |  |  |  |  |  |  |  |  |  |
| 11 DR R | .07 | -.15 | .18 | -.10 | -.05 | .11 | -.25 | -.13 | .53 | .24 | - |  |  |  |  |  |  |  |  |  |  |  |  |  |  |  |
| 12 3BC2 | .02 | .12 | -.08 | -.10 | .05 | .14 | -.11 | .23 | .04 | -.09 | 0 | - |  |  |  |  |  |  |  |  |  |  |  |  |  |  |
| 13 3BC3 | .11 | -.05 | .06 | -.07 | .06 | -.04 | -.25 | .24 | .10 | .29 | .14 | .61 | - |  |  |  |  |  |  |  |  |  |  |  |  |  |
| 14 3BST | .04 | .06 | .02 | -.06 | .03 | -.05 | -.12 | .08 | -.07 | -.18 | -.11 | .28 | .26 | - |  |  |  |  |  |  |  |  |  |  |  |  |
| 15 BRL1 | .15 | -.12 | .06 | .15 | .04 | .06 | -.23 | .21 | 0 | -.05 | -.13 | .14 | .07 | .14 | - |  |  |  |  |  |  |  |  |  |  |  |
| 16 BRL2 | .13 | -.12 | .10 | .02 | -.14 | .05 | -.01 | .10 | .07 | -.09 | -.11 | .24 | -.04 | .06 | .60 | - |  |  |  |  |  |  |  |  |  |  |
| 17 BRL3 | .29 | -.03 | .05 | .06 | -.06 | .09 | .05 | .21 | 0 | -.01 | .02 | -.02 | -.12 | -.05 | .46 | .69 | - |  |  |  |  |  |  |  |  |  |
| 18 BRL4 | .01 | 0 | -.02 | .07 | -.04 | .11 | .13 | .30 | -.02 | .03 | -.17 | .16 | -.11 | .04 | .50 | .60 | .71 | - |  |  |  |  |  |  |  |  |
| 19 BRL5 | .10 | -.07 | .03 | .16 | .07 | .02 | .02 | .23 | .10 | .09 | .07 | .01 | -.01 | -.01 | .60 | .64 | .75 | .57 | - |  |  |  |  |  |  |  |
| 20 BRL6 | .13 | 0 | .06 | -.01 | .06 | -.01 | .01 | .20 | .19 | .12 | .06 | .02 | -.03 | 0 | .41 | .39 | .62 | .66 | .53 | - |  |  |  |  |  |  |
| 21 BRR1 | .12 | -.01 | -.01 | .10 | -.19 | -.06 | .03 | .06 | .07 | -.15 | .05 | .16 | -.07 | .01 | .56 | .43 | .71 | .51 | .40 | .37 | - |  |  |  |  |  |
| 22 BRR2 | -.01 | -.07 | -.14 | .04 | -.03 | -.13 | .03 | .06 | -.25 | -.10 | -.11 | .05 | -.37 | .08 | .46 | .60 | .58 | .44 | .47 | .25 | .68 | - |  |  |  |  |
| 23 BRR3 | .04 | -.02 | -.18 | .04 | -.08 | -.26 | .21 | .21 | -.33 | -.18 | -.12 | .17 | -.23 | .21 | .54 | .55 | .75 | .57 | .41 | .39 | .68 | .85 | - |  |  |  |
| 24 BRR4 | .16 | -.16 | 0 | .16 | -.15 | .04 | .04 | .15 | .19 | -.03 | -.08 | .07 | -.09 | -.01 | .36 | .43 | .53 | .47 | .31 | .11 | .76 | .71 | .76 | - |  |  |
| 25 BRR5 | .08 | .04 | -.06 | .34 | .02 | -.14 | .18 | .25 | -.12 | -.01 | -.07 | .03 | -.30 | -.11 | .47 | .54 | .71 | .66 | .52 | .63 | .60 | .81 | .86 | .72 | - |  |
| 26 BRR6 | -.06 | -.04 | -.08 | .19 | -.14 | -.16 | .11 | .25 | -.24 | -.10 | -.28 | -.13 | -.24 | .01 | .47 | .57 | .63 | .58 | .56 | .28 | .59 | .78 | .83 | .83 | .86 | - |

*Note.* Gen = Gender. SES = Socioeconomic status. Lang = MCDI Total Score. D.O. = Duration of Orienting. D.L. = Distress to Limitations. Attn. = Median Looking Time from Attention Task. Pro = Prohibition Task. DR P = Delayed Response Perseverative Errors (Reversed). DR F = Delayed Response Number of Correct Trials before First Error. DR R = Delayed Response Number of Correct Reversals. 3BC2 = Three Boxes Task Number of Searches to Find Car 2 (Reversed). 3BC3 = Three Boxes Task Number of Searches to Find Car 3. 3BST = Three Boxes Strategy Score. BRL = Ball Run Task Learning Phase. BRR = Ball Run Task Reversal Phase.

Table S2. *Polychoric and Tetrachoric Correlations between Alternative Indicators of Executive Function Task Performance at 14 Months.*

|  |  | 1 | 2 | 3 | 4 | 5 | 6 | 7 |
| --- | --- | --- | --- | --- | --- | --- | --- | --- |
| 1 | Prohibition Task Pass/Fail | - |  |  |  |  |  |  |
| 2 | Ball Run Learning Pass/Fail | .21** | - |  |  |  |  |  |
| 3 | Ball Run Reversal Pass/Fail | .17* | .71** | - |  |  |  |  |
| 4 | Three Boxes Cars Retrieved | .18* | .11 | .01 | - |  |  |  |
| 5 | Delayed Response Reversals | -.09 | -.05 | -.03 | .07 | - |  |  |
| 6 | Ball Run Learning Total | .22** | .87** | .26** | .07 | -.08 | - |  |
| 7 | Ball Run Reversal Total | .11 | .63** | .78** | .03 | -.09 | .54** | - |
|  | Age (Months) | .08 | .18 | -.06 | .01 | .10 | .15 | .05 |
|  | Language | -.02 | .15 | .25** | -.15 | -.07 | .10 | .13 |
|  | Attention at 4 Months | .06 | .01 | .04 | -.13 | -.23* | .01 | .04 |
|  | Duration of Orienting | -.02 | -.06 | .01 | .001 | -.04 | -.02 | -.10 |
|  | Distress to Limitations | -.07 | -.002 | -.03 | .07 | .05 | .05 | -.09 |

*Note.* ***p*<.01. **p*<.05. Ball Run Pass/Fail is based on a score of 4 or more correct trials in each condition. Three Boxes Cars Retrieved is the total number of cars (out of 3) retrieved. Delayed Response Reversals is the number of correct responses in trials where the hiding location is switched following a correct retrieval. Ball Run Totals are the summed number of correct responses in each condition. Language = Parent rating on the MCDI. Attention at 4 Months = Median Looking Time from Attention Task.

Table S3. *WLSMV Estimates for Longitudinal Model Predicting Executive Function Task Performance at 14 Months.*

| **Parameter** | **Unstandardized**  **Estimate (SE)** | **Standardized Estimate** |
| --- | --- | --- |
| **Ball Run Factor BY** |  |  |
| BR Learn 1 | 1.00 | .63*** |
| BR Learn 2 | 1.16 (0.16) | .74*** |
| BR Learn 3 | 1.37 (0.19) | .87*** |
| BR Learn 4 | 1.19 (0.17) | .76*** |
| BR Learn 5 | 1.14 (0.17) | .73*** |
| BR Learn 6 | 0.94 (0.18) | .59*** |
| BR Reversal 1 | 1.18 (0.17) | .75*** |
| BR Reversal 2 | 1.32 (0.18) | .84*** |
| BR Reversal 3 | 1.43 (0.18) | .91*** |
| BR Reversal 4 | 1.22 (0.18) | .77*** |
| BR Reversal 5 | 1.44 (0.19) | .91*** |
| BR Reversal 6 | 1.40 (0.18) | .89*** |
|  |  |  |
| **Delayed Response Factor BY** |  |  |
| DR Correct Reversals | 1.00 | .62*** |
| DR Perseverative (Rev) | 1.38 (0.29) | .85*** |
| DR Trials Before Error | 0.81 (0.17) | .50*** |
|  |  |  |
| **Three Boxes Factor BY** |  |  |
| TB Searches for Car 3 | 1.00 | .72*** |
| TB Searches for Car 2 | 1.17 (0.29) | .84*** |
| TB Strategy | 0.48 (0.13) | .34*** |
|  |  |  |
| **Ball Run Factor ON** |  |  |
| Age | 0.14 (0.09) | .13 |
| Gender | -0.11 (0.11) | -.09 |
| SES | -0.002 (0.01) | -.02 |
| Language | 0.004 (0.003) | .13 |
| 4M Attention | 0.01 (0.01) | .08 |
| 4M Dur. Orient. | -0.07 (0.07) | -.10 |
| 4M Dis. Limit. | -0.02 (0.06) | -.03 |
|  |  |  |
| **Delayed Response Factor ON** |  |  |
| Age | 0.009 (0.10) | .01 |
| Gender | -0.22 (0.12) | -.18 |
| SES | 0.03 (0.01) | .25* |
| Language | 0.005 (0.003) | .14 |
| 4M Attention | -0.05 (0.02) | -.34** |
| 4M Dur. Orient. | -0.05 (0.07) | -.07 |
| 4M Dis. Limit. | 0.06 (0.07) | .09 |
|  |  |  |

*Note.* ^***^*p* < .001. ^**^*p* < .01. ^*^*p*<.05. BY = Factor Loadings. ON = Regressed onto. WITH = Correlated with.

Table S2 (Continued). *WLSMV Estimates for Longitudinal Model Predicting Executive Function Task Performance at 14 Months.*

| **Parameter** | **Unstandardized**  **Estimate (SE)** | **Standardized Estimate** |
| --- | --- | --- |
| **Three Boxes Factor ON** |  |  |
| Age | 0.08 (0.10) | .06 |
| Gender | 0.14 (0.14) | .09 |
| SES | -0.004 (0.01) | -.03 |
| Language | -0.005 (0.004) | -.14 |
| 4M Attention | -0.03 (0.02) | -.18* |
| 4M Dur. Orient. | 0.09 (0.08) | .11 |
| 4M Dis. Limit. | 0.06 (0.08) | .08 |
| **Prohibition ON** |  |  |
| Age | -0.18 (0.15) | -.10 |
| Gender | 0.06 (0.20) | .03 |
| SES | 0.02 (0.02) | .13 |
| Language | 0.002 (0.006) | .04 |
| 4M Attention | -0.02 (0.02) | -.08 |
| 4M Dur. Orient. | 0.04 (0.12) | .03 |
| 4M Dis. Limit. | 0.05 (0.11) | .05 |
| **Prohibition WITH** |  |  |
| Ball Run Factor | 0.15 (0.07) | .24* |
| Delayed Response Factor | -0.08 (0.08) | -.16 |
| Three Boxes Factor | 0.21 (0.09) | .31* |
| **Ball Run Factor WITH** |  |  |
| Delayed Response Factor | -0.03 (0.04) | -.09 |
| Three Boxes Factor | 0.03 (0.05) | .08 |
| **Delayed Response Factor WITH** |  |  |
| Three Boxes Factor | -0.01 (0.05) | -.03 |
| **4M Dur. Orient. WITH** |  |  |
| Age | 0.04 (0.04) | .07 |
| SES | -0.22 (0.36) | -.04 |
| Language | 2.49 (1.24) | .16* |
| 4M Attention | 0.43 (0.28) | .12 |
| 4M Dis. Limit. | -0.04 (0.05) | -.05 |
| **4M Dis. Limit WITH** |  |  |
| Age | 0.03 (0.04) | .06 |
| SES | 0.83 (0.38) | .15* |
| Language | 2.20 (1.34) | .14 |
| 4M Attention | -0.34 (0.28) | -.09 |
| **4M Attention WITH** |  |  |
| Age | -0.11 (0.20) | -.05 |
| SES | 1.22 (1.92) | .05 |
| Language | 6.21 (6.27) | .08 |
| **Age WITH** |  |  |
| SES | 0.39 (0.29) | .11 |
| Language | 1.87 (0.94) | .17 |
| **SES WITH Language** | -7.62 (8.67) | -.07 |

*Note.* ^***^*p* < .001. ^**^*p* < .01. ^*^*p*<.05. BY = Factor Loadings. ON = Regressed onto. WITH = Correlated with.
